# Real-World Efficacy and Safety of Anlotinib With and Without Immunotherapy in Advanced Non-Small Cell Lung Cancer

**DOI:** 10.3389/fonc.2021.659380

**Published:** 2021-07-29

**Authors:** Qi Xiong, Boyu Qin, Lingli Xin, Bo Yang, Qi Song, Yu Wang, Sujie Zhang, Yi Hu

**Affiliations:** ^1^Department of Oncology, General Hospital of Chinese PLA, Beijing, China; ^2^Department of Gynaecology and Obstetrics, People's Liberation Army (PLA) Rocket Force Characteristic Medical Center, Beijing, China

**Keywords:** anlotinib, immunotherapy, anti-programmed death-1/programmed death-ligand 1, non-small cell lung cancer, target therapy

## Abstract

**Aims:**

Combination of anti-angiogenesis therapy and immunotherapy has showed synergistic effects in non-small cell lung cancer (NSCLC). The aim of this retrospective study was to investigate the efficacy and safety of anlotinib with and without immunotherapy in NSCLC.

**Methods:**

Pathologically confirmed NSCLC patients (stage IIIB-IV) receiving anlotinib between November 2018 and February 2020 were enrolled for retrospective analysis. The outcomes and safety of overall patients were evaluated, and the efficacies of anlotinib plus immunotherapy and anlotinib alone was compared. The primary endpoint was progression-free survival (PFS).

**Results:**

A total of 80 patients (median age: 62 years, range: 29-86 years) were included. Overall median PFS was 4.3 months (95% confidence interval (CI): 2.7-5.9 months). In univariate analysis, patients without *EGFR* mutation, previous EGFR target therapy, and brain metastasis had significantly longer PFS. Cox regression analysis showed that only brain metastasis was an independent predictor of PFS. The median PFS of patients receiving anlotinib plus immunotherapy was slightly longer than that of patients receiving anlotinib alone (4.2 *vs* 3.1 months); however, the difference was not statistically significant. A tendency of longer median PFS was observed in patients with adenocarcinoma, *EGFR* wild type, stage IV, no liver metastasis, former smoker, ≥2 previous treatment lines, no previous VEGF or EGFR target therapies in anlotinib plus immunotherapy group. Treatments with anlotinib alone or anlotinib plus immunotherapy were well tolerable. The most common adverse events were fatigue, decreased hemoglobin count, hypertension, hand-foot syndrome, oral mucositis and hoarseness.

**Conclusion:**

Anlotinib is well tolerable and effective in advanced NSCLC patients. Brain metastasis is an independent predictor of PFS in NSCLC patients receiving anlotinib. Future prospective studies with larger sample size and extended follow-up are needed to confirm the clinical benefit in NSCLC patients treated with anlotinib combined with immunotherapy.

## Introduction

Lung cancer is the most commonly diagnosed cancer and the leading cause of cancer-related mortality worldwide (1, 2). About 80%–85% of patients have non-small cell lung cancers (NSCLC), and most have locally advanced or metastatic disease at diagnosis and therefore poor prognosis. Target therapy and immunotherapy have remarkably improved the overall survival (OS) and quality of life of these patients (3–7); however, for those who fail frontline treatment but remain in satisfactory physical condition, treatment options are limited. Novel effective drugs need to be identified.

Anlotinib is a tyrosine kinase inhibitor (TKI) targeting multiple tumor angiogenesis and proliferation signaling receptors, including receptor tyrosine kinases vascular endothelial growth factor receptor 1-3 (VEGFR 1-3), epidermal growth factor receptor (EGFR), fibroblast growth factor receptor 1-4 (FGFR 1-4), platelet-derived growth factor receptor α and β (PDGFR α and β), and stem cell factor receptor (c-Kit) (8–10).The phase III ALTER 0303 study demonstrated that patients with advanced NSCLC receiving anlotinib as third or further line treatment had significantly longer OS (median OS: 9.6 *vs* 6.3 months) and progression-free survival (PFS) (median PFS: 5.4 *vs* 1.4 months) than patients receiving placebo (11). Based on these findings, anlotinib was approved as third line therapy for advanced NSCLC patients after two lines of chemotherapy by the China National Medical Products Administration in 2018. In a real-world study, Wu et al. retrospectively analyzed the efficacy of anlotinib as salvage treatment in 81 NSCLC patients and reported similar results: the median PFS of NSCLC patients treated with anlotinib was 5 months, with patients with Eastern Cooperative Oncology Group performance status (ECOG PS) 0-1 and those without brain metastasis most likely achieve longer PFS (12). In another study of 52 NSCLC patients receiving anlotinib as third- or later-line treatment, Zhang et al. found median PFS and OS to be 4.5 months and 9 months, respectively, with relatively longer survival rates in patients with ECOG PS 0–1 and patients without liver metastasis (13). In contrast to these findings, Shao et al. reported median PFS of only 3.3 months in 58 advanced lung cancer patients (50 with NSCLC) who were treated with anlotinib as third or later-line treatment (14). These inconsistent results call for more detailed analysis of the real-world efficacy of anlotinib.

Previous studies have shown that combination of anti-angiogenesis therapy and other type of therapies had synergistic effects, and revealed prolonged PFS and OS in the first line and further line treatment (15–17). However, previous clinical trials failed to demonstrate a survival benefit with combination of anti-angiogenesis TKIs (such as nintedanib, sorafenib, and sunitinib) with chemotherapy in NSCLC (18–21). At the 20th World Conference on Lung Cancer (WCLC 2019), Han and colleagues reported the results of phase II clinical trials which showed that the combination of anlotinib and chemotherapy or target therapy as first-line treatment for advanced NSCLC increased the objective response rate (ORR) and the disease control rate (DCR): ORR was 60% for chemotherapy plus anlotinib and 92.6% for erlotinib plus anlotinib, while DCR was 96.7% for chemotherapy plus anlotinib and 100% for erlotinib plus anlotinib (22, 23). In addition, in a phase I study, Han et al. reported ORR of 72.7%, DCR of 100%, and 12-month PFS rate of 71.4% with the combination of sintilimab with anlotinib as first-line treatment for NSCLC (24, 25). These evidences suggest that anlotinib combined with other therapies might be promising treatment for advanced NSCLC. Indeed, these therapeutic strategies have already been applied in clinical practice. Wang et al. showed that the median PFS and OS were 6.9 months and 14.5 months in 67 NSCLC patients treated with anlotinib plus anti-programmed death-1 (PD-1) antibody after previous systemic treatment (26).Thus, we performed a retrospective analysis of real-world data to investigate the efficacy of anlotinib used alone and in combination with other agents in advanced NSCLC, with special attention to the combination of anlotinib with immunotherapy.

## Patients and Methods

### Patients and Treatments

Pathologically confirmed NSCLC patients (stage IIIB-IV according to AJCC 8th edition cancer staging system) receiving anlotinib in General Hospital of Chinese PLA between November 2018 and February 2020 were eligible for retrospective analysis. The inclusion criteria were as follows: advanced stage (IIIB-IV) NSCLC confirmed pathologically; with at least one measurable lesion; treated with anlotinib for the first time regardless of combined therapy and treatment lines; aged over 18 years old; ECOG PS score 0-2; radiotherapy allowed for stage III patients. The exclusion criteria were as follows: previous use of anlotinib; diagnosis with concomitant other cancers other than NSCLC; patients with hemoptysis (>50 ml/d) or deep vein thrombosis or pulmonary embolism; patients participated in clinical trials of other anti-tumor drugs within four weeks; severe disfunction of heart, liver, kidney and (or) other complications that might detrimental for survival determined by investigators. The medical records of the patients were reviewed to collect data on sex, age, pathological type, stage, presence of liver/brain metastasis, ECOG PS, smoking history, previous treatments, and status of *EGFR*, *ROS1*, *ALK*, *HER2, TP53*, and *KRAS*.

Anlotinib was administered once daily (12mg or 10 mg or 8 mg) on days 1–14 of a 21-day cycle, or every other day. The starting dose of anlotinib was determined by the oncologist according to the patients’ status. Immunotherapy was with anti-PD-1 or programmed death-ligand 1 (PD-L1) antibodies, and included pembrolizumab (2 mg/kg every 3 weeks), nivolumab (3 mg/kg every 2 weeks), sintilimab (200 mg every 3 weeks), toripalimab (240 mg every 3 weeks), or atezolizumab (1200 mg every 3 weeks). Follow-up data were collected up to April 30th, 2020.

This study was approved by Ethics Committee of the General Hospital of Chinese PLA (Medical Ethics Committee of General Hospital of Chinese PLA No. S2018-092-01) and conducted according to the principles of the Declaration of Helsinki. The requirement for informed consent was waived in view of the retrospective nature of the study.

### Outcomes and Safety Evaluation

Therapeutic effect was assessed using Response Evaluation Criteria in Solid Tumors (RECIST) version 1.1 by computed tomography (CT) scans every 2 cycles by two doctors independently and categorized as complete response (CR), partial response (PR), stable disease (SD), or progressive disease (PD). When there is disagreement on CT evaluation, a third doctor was requested to reevaluate. PFS was defined as the time from anlotinib initiation until disease progression or death of any cause before disease progression. OS was defined as the time from the beginning of anlotinib to death. Best response was used to calculate objective response rate (ORR) and disease control rate (DCR). Treatment-related adverse events were graded using Common Terminology Criteria for Adverse Events (CTCAE) version 5.0.

### Statistical Analysis

The chi-square test and Fisher’s exact test were used to compare the baseline characteristics and treatment response between two groups. Analysis of variance (ANOVA) was used to compare the difference among three or more groups. Survival curves for OS and PFS were analyzed using the Kaplan–Meier method. The log-rank test was used for univariate analysis between groups. Cox regression was used to analyze the statistically significant factors according to results of univariate analysis. Forest plots were used to depict the results of stratified analysis. Statistical analysis was performed by PRISM version 7.0 (GraphPad Software, La Jolla, CA, USA) and SPSS version 26.0 (IBM Corp., Armonk, NY, USA). P<0.05 (two-sided) was defined as statistical significance.

## Results

### Baseline Clinical Characteristics of Patients

A total of 80 patients were included in our study. There were 59 (73.7%) males and 21 (26.3%) females. The median age of the patients was 63 years (range, 29 to 86 years). While 12 of 80 (15%) patients staged IIIB/IIIC, 68 (85%) patients staged IV. Pathological types included adenocarcinoma (55/80, 68.7%) squamous cell carcinoma (22/80, 27.5%), and unknown type NSCLC (3/80, 3.8%). 13 (16.3%) patients had liver metastasis and 20 (25.0%) patients had brain metastasis. *EGFR* mutation was identified in 21/80 (26.3%) patients, *TP53* mutation in 13/80 (16.3%) patients, *KRAS* mutation in 5/80 (6.3%) patients, and *ALK* mutation in 2/80 (2.5%) patients. While 5 (6.2%) patients received anlotinib in first-line treatment, 25 (31.3%) received anlotinib in second-line treatment, and 50 (62.5%) received anlotinib in third- or later-line treatment. Anlotinib was used alone in 24/80 (30.0%) patients and in combination with other therapies in 56/80 (70%) patients; among the latter, 30 patients received anlotinib plus immunotherapy, 9 received anlotinib plus TKIs target therapy, 11 received anlotinib plus chemotherapy, and 6 received anlotinib plus immunotherapy and chemotherapy. **Table 1** summarizes the characteristics of the patients.

**Table 1:** Characteristics of patients and univariate analysis of factors associated with progression–free survival in the entire sample.

Characteristics	Patients (*n*= 80)	mPFS (95%CI)	*P* value	HR (95% CI)
**Sex, n (%)**			0.227	1.437 (0.791-2.611)
Male	59 (73.7)	5.1 (2.4-7.8)		
Female	21 (26.3)	3.3 (2.9-3.7)		
**Age, n (%)**				
			0.997	1.001 (0.571-1.753)
≤65	50 (62.5)	4.2 (2.1-6.3)		
>65	30 (37.5)	4.3 (1.7-6.9)		
**Pathologic type, n (%)**			0.363	0.907 (0.546-1.506)
Adenocarcinoma	55 (68.7)	3.0 (2.2-3.8)		
Squamous cell carcinoma	22 (27.5)	6.0 (4.4-7.6)		
Unknown	3 (3.8)	4.1 (0.0-9.4)		
**EGFR status, n (%)**			0.011*	0.478 (0.265-0.861)
Mutation	21 (26.3)	2.8 (2.2-3.4)		
Wildtype/Unknown	59 (73.7)	6.0 (3.5-8.5)		
**TP53 status, n (%)**			0.950	1.025 (0.480-2.188)
Mutation	13 (16.3)	4.6 (3.8-5.4)		
Wild type/Unknown	67 (83.7)	4.3 (2.2-6.4)		
**KRAS status, n (%)**			0.555	0.706 (0.218-2.281)
Mutation	5 (6.3)	2.7 (0.1-5.3)		
Wild type/Unknown	75 (93.7)	4.3 (2.5-6.1)		
**Clinical stage, n (%)**			0.143	1.863 (0.795-4.366)
IIIB/IIIC	12 (15.0)	7.8 (5.3-10.4)		
IV	68 (85.0)	3.3 (1.4-5.2)		
**Liver metastases, n (%)**			0.707	1.148 (0.557-2.363)
No	67 (83.7)	4.3 (2.5-6.1)		
Yes	13 (16.3)	3.0 (0.0-6.6)		
**Brain metastases, n (%)**			0.025*	1.924 (1.069-3.463)
No	60 (75.0)	5.1 (3.4-6.8)		
Yes	20 (25.0)	2.3 (1.2-3.4)		
**Smoking history, n (%)**			0.612	0.925 (0.681-1.257)
Never smoked	32 (40.0)	4.1 (2.1-6.1)		
Current smoker	19 (23.7)	3.3 (0.8-5.8)		
Former smoker	29 (36.3)	7.1 (2.8-11.4)		
**ECOG PS, n (%)**			0.434	1.372 (0.615-3.060)
≤1	70 (87.5)	4.6 (2.7-6.5)		
>1	10 (12.5)	1.9 (0.0-3.8		
**No. of previous treatment lines, n (%)**			0.516	1.207 (0.680-2.144)
<2	30 (37.5)	5.4 (1.1-9.7)		
≥2	50 (62.5)	4.2 (2.1-6.3)		
**Previous VEGF-target therapy, n (%)**			0.298	1.331 (0.772-2.294)
No	45 (56.3)	5.4 (3.0-7.8)		
Yes	35 (43.7)	3.3 (1.4-5.2)		
**Previous EGFR-target therapy, n (%)**			0.040*	1.844 (1.013-.3.357)
No	61 (76.2)	5.4 (2.9-7.9)		
Yes	19 (23.8)	2.8 (1.8-3.8)		
**Combination therapy, n (%)**			0.784	0.971 (0.767-1.228)
No	24 (30.0)	2.8 (0.6-5.0)		
immunotherapy	30 (37.5)	4.1 (0.0-11.2)		
chemotherapy	11 (13.7)	5.4 (1.1-9.7)		
targeted therapy	9 (11.3)	3.1(1.1-5.1)		
immunotherapy and chemotherapy	6 (7.5)	4.2 (1.8-6.6)		

Data were present as n (%) unless specified.

CI, confidence interval; ECOG PS, indicates Eastern Cooperative Oncology Group Performance Status (score range: 0-5, with the highest score indicating death); EGFR, endothelial growth factor receptor; HR, hazard ratio; PFS, progression-free survival; VEGF, vascular endothelial growth factor. The bold value with symbol * indicates statistically significant.

### Overall Efficacy of Treatment

**Figure 1:**
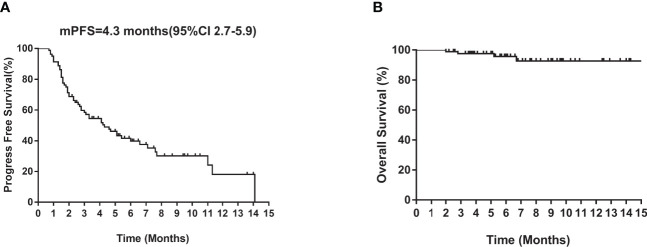
Kaplan–Meier curves of median **(A)** PFS and **(B)** OS of all patients. CI, confidence interval; PFS, progression-free survival; OS, overall survival.

**Figure 2:**
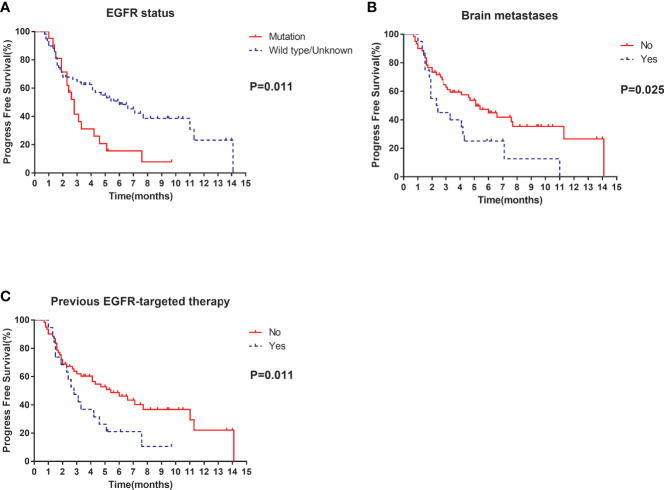
Univariate analysis of PFS in all patients: **(A)** stratified by *EGFR* status; **(B)** stratified by brain metastases; **(C)** stratified by previous EGFR-targeted therapy.

Of the 80 patients, PR was achieved in 9/80 patients, SD in 49/80 patients, and PD in 22/80 patients. The overall ORR and DCR were 11.3% and 72.5%, respectively. The median follow-up duration was 6.1 months (range: 2.0–15.1 months). Treatment with anlotinib was still ongoing for 27 patients by the day of last follow-up. Overall median PFS was 4.3 months (95% confidence interval (CI): 2.7-5.9 months) (**Figure 1A**). Univariate analysis revealed that patients without *EGFR* mutation, previous EGFR target therapy and brain metastasis had statistically significant longer PFS (**Figures 2A–C** and **Table 1**). Age, sex, pathological type, *TP53* and *KRAS* status, stage, liver metastasis, smoking history, ECOG PS, and previous treatment lines or regimens were not associated with PFS (**Table 1**). In multivariate cox regression analysis, brain metastasis was the only factor independently associated with PFS (HR: 1.816, 95%CI: 1.003-3.286, P=0.049) (**Table 2**). *EGFR* mutation and previous EGFR target therapy were not independent risk factors for PFS. The 1-year survival rate was 92.6% in this study. Since 76 (95%) patients were still alive at last follow-up, OS was not analyzed (**Figure 1B**).

**Table 2:** Cox regression analysis of factors associated with progression-free survival in the entire sample.

Factor	*p* value	HR	HR95%CI	
			Lower	Upper
EGFR status	0.184	0.466	0.151	1.438
Brain metastases	0.049*	1.816	1.003	3.286
Previous EGFR-target therapy	0.880	0.916	0.293	2.865

CI, confidence interval; EGFR, endothelial growth factor receptor; HR, hazard ratio. The bold value with symbol * indicates statistically significant.

### Efficacy of Anlotinib Combined With Immunotherapy

To explore whether immunotherapy could improve the efficacy of anlotinb, we compared the efficacy of anlotinib plus immunotherapy (anti-PD-1 or PD-L1 antibodies) with anlotinib alone in NSCLC. There were 30 patients receiving anlotinib combined with immunotherapy, and 24 receiving anlotinib alone. Baseline characteristics were comparable between the two groups (**Table 3**).

**Table 3:** Comparison of clinical characteristics of patients receiving anlotinib alone and those receiving anlotinib plus immunotherapy.

Characteristics	Anlotinib alone group (*n*=24)	Anlotinib plus immunotherapy group(*n*=30)	*p* value
**Sex, n (%)**			0.380
Male	20 (83.3)	22 (73.3)	
Female	4 (16.7)	8 (26.7)	
**Age, n (%)**			0.124
≤65	11 (45.8)	20 (66.7)	
>65	13 (54.2)	10 (33.3)	
**Pathologic type, n (%)**			
Adenocarcinoma	13 (54.2)	22 (73.3)	
Squamous cell carcinoma	11 (45.8)	6 (20.0)	
Unknown	0 (0)	2 (6.7)	
**EGFR status, n (%)**			0.713
Mutation	5 (20.83)	4 (13.3)	
Wild type/Unknown	19 (79.17)	26 (86.7)	
**TP53 status, n (%)**			0.309
Mutation	1 (4.2)	5 (16.7)	
Wild type/Unknown	23 (95.8)	25 (83.3)	
**KRAS status, n (%)**			1.000
Mutation	2 (8.3)	3 (10.0)	
Wild type/Unknown	22 (91.7)	27 (90.0)	
**Clinical stage, n (%)**			0.966
IIIB/IIIC	3 (12.5)	5 (16.7)	
IV	21 (87.5)	25 (83.3)	
**Liver metastases, n (%)**			0.678
No	18 (75.0)	25 (83.3)	
Yes	6 (25.0)	5 (16.7)	
**Brain metastases, n (%)**			0.066
No	23 (95.8)	22 (73.3)	
Yes	1 (4.2)	8 (26.7)	
**Smoking history, n (%)**			0.091
Never smoked	7 (29.2)	10 (33.3)	
Current smoker	5 (20.8)	13 (43.4)	
Former smoker	12 (50.00)	7 (23.3)	
**ECOG PS, n (%)**			0.257
≤1	19 (79.2)	28 (93.3)	
>1	5 (20.8)	2 (6.7)	
**No. of previous treatment lines, n (%)**			0.614
<2	8 (33.3)	12 (40.0)	
≥2	16 (66.7)	18 (60.0)	
**Previous VEGF-target therapy, n (%)**			0.061
No	18 (75.0)	15 (50.0)	
Yes	6 (25.0)	15 (50.0)	
**Previous EGFR-target therapy, n (%)**			0.713
No	19 (79.2)	26 (86.7)	
Yes	5 (20.8)	4 (13.3)	

**Figure 3:**
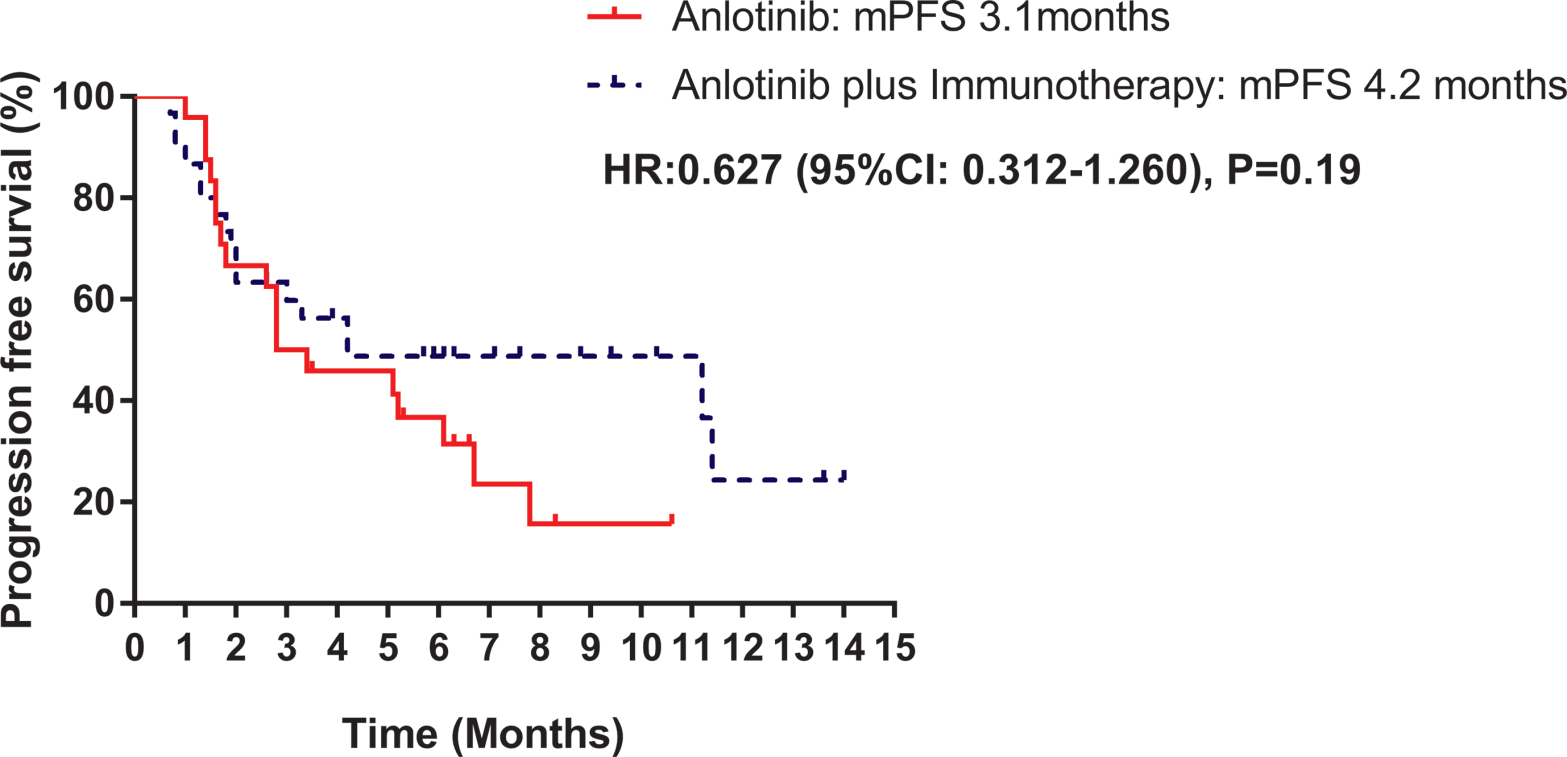
Kaplan–Meier curves of PFS between anlotinib arm and anlotinib plus immunotherapy arm.

**Figure 4:**
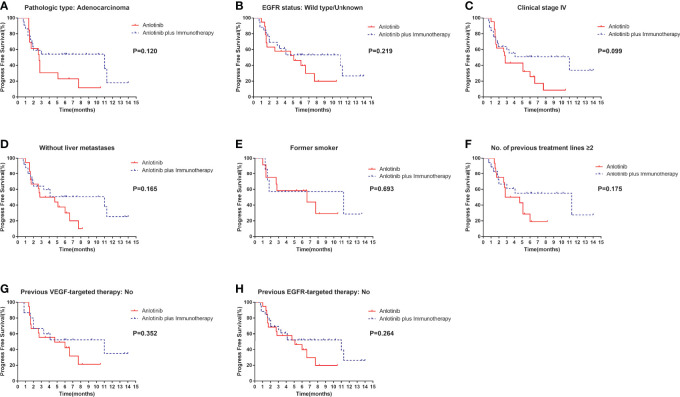
Stratification analysis of PFS between patients in the anlotinib arm and anlotinib plus immunotherapy arm. **(A)** stratified by pathologic type: Adenocarcinoma; **(B)** stratified by *EGFR* status: Wild type/Unknown; **(C)** stratified by clinical stage IV; **(D)** stratified by without liver metastases; **(E)** stratified by former smoker; **(F)** stratified by No. of previous treatment lines ≥2; **(G)** stratified by previous VEGF-targeted therapy: No; **(H)** stratified by previous EGFR-targeted therapy: No.

**Figure 5:**
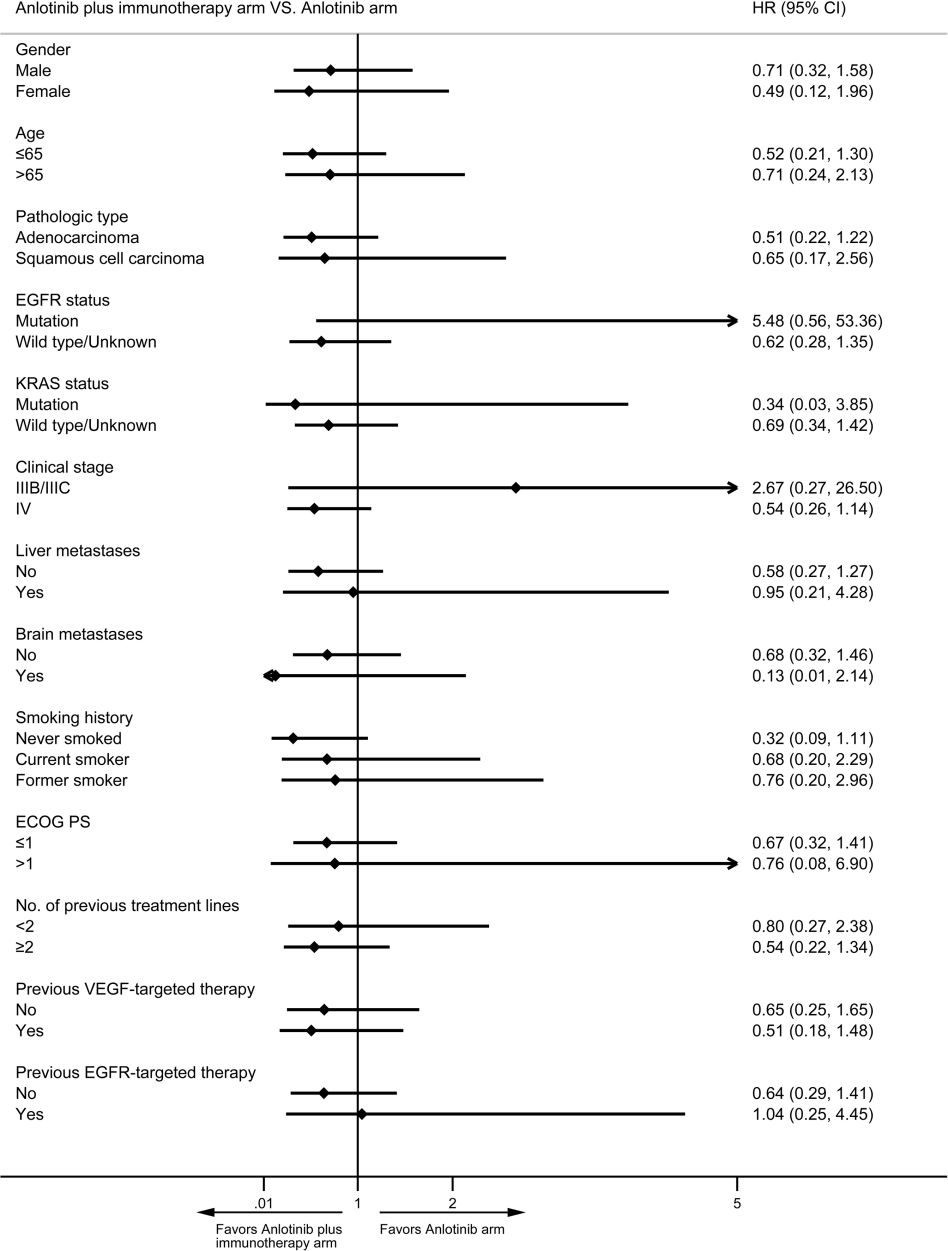
Forest plot of stratification analysis of PFS between patients in anlotinib arm and anlotinib plus immunotherapy arm.

Median PFS was longer for patients receiving anlotinib plus immunotherapy than for patients receiving anlotinib alone, although the difference was not statistically significant (4.2 months *vs*. 3.1 months; HR: 0.627, 95% CI: 0.312–1.260; P = 0.19) (**Figure 3**). The ORR (12.5% *vs* 10%) and DCR (70.8% *vs* 70%) were similar between anlotinib arm and anlotinib combined with immunotherapy arm (**Table 4**). Stratified analysis was performed to identify the subgroups most likely to benefit from the addition of immunotherapy to anlotinib therapy. Although no statistically significant differences were found, median PFS tended to be longer in patients with adenocarcinoma, *EGFR* wild type, stage IV, no liver metastasis, former smoker, ≥2 previous treatment lines, no previous VEGF or EGFR target therapies (**Table 5** and **Figures 4A–H**). **Figure 5** presents a forest plot of the stratified analysis.

**Table 4:** Response of anlotinib alone and anlotinib plus immunotherapy.

	Anlotinib alone group(*n*=24)	Anlotinib plus immunotherapy group (*n*=30)	*p* value
CR	0	0	
PR	3	3	
SD	14	18	
PD	7	9	
ORR (%)	3 (12.5%)	3 (10.0%)	1.000
DCR (%)	17 (70.8%)	21 (70.0%)	0.947

CR, complete response; DCR, disease control rate; ORR, objective response rate; PD, progressive disease; PR, partial response; SD, stable disease.

**Table 5:** Comparison of treatment efficacy between patients treated with anlotinib alone and patients treated with anlotinib plus immunotherapy.

Variable	Total patients (*n*=54)	mPFS (95%CI)		*p* value	HR (95% CI)
		Anlotinib alone group (*n*=24)	Anlotinib plus immunotherapy group (*n*=30)		
**Sex, n (%)**					
Male	42 (77.8)	2.8 (0.0-7.0)	4.1 (0.0-11.3)	0.400	0.715 (0.324-1.578)
Female	12 (22.2)	2.8 (0.000-6.3)	4.1(2.1-6.1)	0.302	0.487 (0.121-1.962)
**Age, n (%)**					
≤65	31 (57.4)	2.7 (1.5-3.9)	4.1 (0.0-10.7)	0.154	0.524 (0.211-1.300)
>65	23 (42.6)	5.1 (1.8-8.4)	–	0.534	0.709 (0.236-2.129)
**Pathologic type, n (%)**					
Adenocarcinoma	35 (64.8)	2.7 (1.7-3.7)	11.0 (0.0-23.6)	0.120	0.514 (0.217-1.216)
Squamous cell carcinoma	17 (31.5)	6.0 (4.226-7.8)	–	0.534	0.652 (0.166-2.563)
Unknown	2 (3.7)				
**EGFR status, n (%)**					
Mutation	9 (16.7)	2.8 (2.6-3.0)	1.3 (0.4-2. 2)	0.101	5.477 (0.562-53.358)
Wild type/Unknown	45 (83.3)	5.1 (1.0-9.2)	11.0 (1.4-20.6)	0.219	0.616 (0.280-1.353)
**TP53 status, n (%)**					
Mutation	6 (11.1)	–	–	0.466	
Wild type/Unknown	48 (88.9)	2.8 (0.0-5.8)	4.1 (0.0-11.9)	0.263	0.666 (0.322-1.375)
**KRAS status, n (%)**					
Mutation	5 (9.3)	–	–	0.364	0.342 (0.030-3.851)
Wild type/Unknown	49 (90.7)	4.7 (2.5-6.9)	4.1(0.011.6)	0.311	0.693 (0.337-1.424)
**Clinical stage, n (%)**					
IIIB/IIIC	8 (14.8)	–	4.1 (0.0-8.6)	0.368	2.667 (0.268-26.499)
IV	46 (85.2)	2.8 (2.5-3.1)	11.0 (0.2-21.8)	0.099	0.543 (0.258-1.143)
**Liver metastases, n (%)**					
No	43 (79.6)	2.8 (0.0-6.7)	11.0 (1.7-20.3)	0.165	0.584 (0.269-1.267)
Yes	11 (20.4)	2.8 (0.0-7.2)	3.0 (0.9-5.1)	0.949	0.952 (0.212-4.279)
**Brain metastases, n (%)**					
No	45 (83.3)	4.7(2.5-6.9)	4.1 (0.0-10.8)	0.311	0.679 (0.317-1.455)
Yes	9 (16.7)	–	4.1 (0.0-9.1)	0.094	0.134 (0.008-2.140)
**Smoking history, n (%)**					
Never smoked	17 (31.5)	2.8 (2.3-3.3)	–	0.053	0.319 (0.092-1.107)
Current smoker	18 (33.3)	1.7 (1.5-1.9)	3.3 (0.8-5.8)	0.519	0.676 (0.200-2.291)
Former smoker	19 (35.2)	6.6 (0.0-15.0)	11.3 (0.0-25.8)	0.693	0.763 (0.197-2.957)
**ECOG PS, n (%)**					
≤1	47 (87.0)	5.1 (2.6-7.6)	4.1 (0.0-10.1)	0.289	0.674 (0.321-1.415)
>1	7 (13.0)	2.7 (0.1-5.3)	–	0.805	0.758 (0.083-6.904)
**No. of previous treatment lines, n (%)**					
<2	20 (37.0)	1.6 (0.0-7.7)	3.3 (0.2-6.4)	0.683	0.798 (0.267-2.384)
≥2	34 (63.0)	2.8 (0.3-5.3)	11.3 (0.9-21.7)	0.175	0.543 (0.221-1.336)
**Previous VEGF-target therapy, n (%)**					
No	33 (61.1)	4.7 (0.0-10.5)	11.0 (0.9-21.1)	0.352	0.647 (0.254-1.648)
Yes	21 (38.9)	2.8 (1.2-4.4)	4.1 (0.3-7.9)	0.196	0.511 (0.177-1.480)
**Previous EGFR-target therapy, n (%)**					
No	45 (83.3)	5.1 (1.1-9.1)	11.0 (1.6-20.4)	0.264	0.643 (0.293-1.412)
Yes	9 (16.7)	2.6 (0.2-5.0)	1.3 (0.4-2.2)	0.954	1.044 (0.245-4.451)

–, not involved in the multivariate analysis.

Data were present as n (%) unless specified.

CI, confidence interval; ECOG PS, indicates Eastern Cooperative Oncology Group Performance Status (score range: 0-5, with the highest score indicating death); EGFR, endothelial growth factor receptor; HR, hazard ratio; PFS, progression-free survival; VEGF, vascular endothelial growth factor.

### Safety Analysis

Treatment-related adverse events occurred in 79 of the 80 patients. The five most common adverse events (all grades) were fatigue (62.5%), decreased hemoglobin count (42.5%), hypertension (41.5%), hand-foot syndrome (37.5%), and oral mucositis (33.75%). The five most common grade 3-5 adverse events were hypertension (13.7%), oral mucositis (8.75%), fatigue (6.25%), hand-foot syndrome (2.5%), liver dysfunction or increased AST (2.5%), hoarseness (1.25%), nausea (1.25%), decreased leukocyte count (1.25%) and hemoptysis (1.25%) (**Table 6**). Discontinuation of treatment due to adverse events was necessary in 6 patients (one patient each for hemoptysis, for grade 4 hypertension, for grade 3 fatigue, for alimentary tract hemorrhage, for grade 3 liver dysfunction, and for edema). There were no treatment-related deaths or life-threatening adverse events.

**Table 6:** Adverse events in patients treated with anlotinib *vs*. patients treated with anlotinib plus immunotherapy.

Adverse event	All Patients (*n*=80)		Anlotinib (*n*=24)		Anlotinib plus Immunotherapy (*n*=30)	
	Any grade	≥3 grade	Any grade	≥3 grade	Any grade	≥3 grade
Fatigue	50 (62.5)	5 (6.3)	13 (54.2)	1 (4.2)	21 (70.0)	4 (13.3)
Decreased hemoglobin count	34 (42.5)	0 (0)	13 (54.2)	0 (0)	11 (36.7)	0 (0)
Hypertension	33 (41.3)	11 (13.7)	9 (37.5)	4 (16.7)	15 (50.0)	6 (20.0)
Hand-foot syndrome	30 (37.5)	2 (2.5)	9 (37.5)	1 (4.2)	12 (40.0)	0 (0)
Oral mucositis	27 (33.8)	7 (8.8)	11 (45.8)	2 (8.3)	10 (33.3)	4 (13.3)
Hoarseness	25 (31.3)	1 (1.3)	6 (25.)	0 (0)	11 (36.7)	1 (3.3)
Nausea	23 (28.8)	1 (1.3)	8 (33.3)	1 (4.2)	8 (26.7)	0 (0)
Rash	20 (25.0)	0 (0)	5 (20.8)	0 (0)	7 (23.3)	0 (0)
Liver dysfunction	18 (22.5)	2 (2.5)	2 (8.3)	0 (0)	9 (30.0)	2 (6.7)
Increased AST	17 (21.3)	2 (2.5)	2 (8.3)	0 (0)	9 (30.0)	2 (6.7)
Diarrhea	15 (18.8)	0 (0)	6 (25.0)	0 (0)	5 (16.7)	0 (0)
Dizziness	12 (15.0)	0 (0)	2 (8.3)	0 (0)	6 (20.0)	0 (0)
Increased ALT	11 (13.8)	0 (0)	0 (0)	0 (0)	6 (20.0)	0 (0)
Proteinuria	10 (12.5)	0 (0)	2 (8.3)	0 (0)	3 (10.0)	0 (0)
Decreased leukocyte count	10 (12.5)	1 (1.3)	1 (4.2)	0 (0)	3 (10.0)	0 (0)
Thrombocytopenia	8 (10.0)	0 (0)	1 (4.2)	0 (0)	3 (10.0)	0 (0)
Increased Scr	8 (10.0)	0 (0)	1 (4.2)	0 (0)	4 (13.3)	0 (0)
Hemoptysis	5 (6.3)	1 (1.3)	3 (12.5)	0 (0)	1 (3.3)	0 (0)
Alimentary tract hemorrhage	5 (6.3)	0 (0)	2 (8.3)	0 (0)	2 (6.7)	0 (0)
Hemorrhinia	2 (2.5)	0 (0)	0 (0)	0 (0)	1 (3.3)	0 (0)

AST, aspartate aminotransferase; ALT, alanine aminotransferase; Scr, serum creatinine.

Among patients receiving anlotinib plus immunotherapy, similar to overall patients, the five most common adverse events were fatigue (70%), hypertension (50%), hand-foot syndrome (40%), decreased hemoglobin level (36.67%), and hoarseness (36.7%). Grade 3–5 adverse events occurred in 14 patients. Among patients receiving anlotinib alone, the five most common adverse events were fatigue (54.17%), decreased hemoglobin level (54.17%), oral mucositis (45.83%), hypertension (37.5%), and hand-foot syndrome (37.5%). Grade 3–5 adverse events occurred in 8 patients. Adverse events were similar in patients treated with anlotinib plus immunotherapy arm and patients treated with anlotinib alone. No additional adverse event was reported in anlotinib plus immunotherapy arm compared to anlotinib arm (**Table 6**).

## Discussion

The ALTER 0303 study and previous real-world studies showed that anlotinib alone could improve prognosis in NSCLC patients as third or later line treatment. Furthermore, the combination of anlotinib with immunotherapy, target therapy, or chemotherapy has been shown to have synergetic efficacy (22–24, 27). We retrospectively analyzed real-world data to determine the efficacies of these therapeutic strategies in NSCLC, paying special attention to the combination of anlotinib plus immunotherapy.

During the course of cancer, about 40% NSCLC patients will finally develop brain metastases, which leads to poor prognosis (28). In this study, brain metastasis was identified as an independent prognostic factor in NSCLC patients treated with anlotinib. Subgroup *post hoc* analysis of ALTER0303 showed similar median PFS in NSCLC patients with and without brain metastases (4.17 *vs* 4.53 months, P=0.69) (29). Zhang et al. also reported brain metastases had no influence on PFS of NSCLC patients treated with anlotinib alone (13). However, in line with wu et al. (12), we found significantly longer median PFS in patients without brain metastases than in patients with brain metastases, whether treated with anlotinib alone or with anlotinib plus other therapies. The differences between ours and previous studies were likely due to differences in treatment regimens and sample sizes; the difference between *post hoc* analysis and retrospective analysis may also contribute.

In the present study, univariate analysis showed significantly longer PFS in patients without EGFR mutation than in patients with the mutation. However, consistent with previous studies (12, 13, 30), cox regression analysis showed both *EGFR* status and previous EGFR target therapy were not independent risk factors for PFS in patients receiving anlotinib. EGFR-TKIs are standard first-line treatment for NSCLC patients with *EGFR* mutations. Unfortunately, patients who show response invariably develop resistance after 8–12 months. The main acquired resistance mechanisms appear to be secondary mutations of *EGFR* (T790M), *KRAS*, *PIK3CA* and *MET* amplification. A recent study suggested that anlotinib could overcome acquired resistance to EGFR-TKI *via* inhibiting FGFR1 (31). A preclinical study showed that anlotinib could inhibit proliferation and induce apoptosis of *KRAS* mutation lung cancer cells through downregulating MEK and ERK. The study also revealed prolonged survival of *KRAS* mutation in lung cancer animal model (32). In addition, there is a previous report of an NSCLC patient harboring *KRAS* mutation who had PFS of 21 months after treatment with anlotinib (33). The mechanisms described above may partially explain why patients with acquired resistance to EGFR-TKIs could benefit from anlotinib. Overall, our results and the evidence from earlier researches indicate that patients can benefit from anlotinib treatment, regardless of *EGFR* status and previous EGFR target therapy.

Immune checkpoint inhibitors targeting PD-1 or PD-L1 have remarkably improved survival in NSCLC patients. However, only about 20% NSCLC patients respond to anti-PD-1/PD-L1 antibody monotherapy. The combination of anti-angiogenesis agents and anti-PD-1/PD-L1 antibody has shown promising benefits in solid tumors such as hepatic carcinoma, renal cancer, and NSCLC (34). Yang et al. reported that anlotinib and anti-PD-1/PD-L1 antibody acted synergistically to provide therapeutic benefit by promoting infiltration of natural killer cells, M1-like tumor-associated macrophages (TAM), and dendritic cells, while reducing the infiltration of M2-like TAM (35). Another study showed that anlotinib reduced PD-L1 expression on vascular endothelial cells *via* inactivation of AKT pathway, which then leads to increase in the CD8/FoxP3 ratio in the tumor immune microenvironment (36). It appears that anlotinib changes the immunosuppressive tumor microenvironment to an immune permissive status, and thus enhances efficacy of PD-1/PD-L1 antibody. However, in the present study, PFS was only slightly longer in patients receiving anlotinib plus anti-PD-1/PD-L1 group than in those receiving anlotinib alone, and no statistical difference was found. It is worth noting that the benefits of the combination of anlotinib and immunotherapy were most noticeable in patients with wild-type *EGFR*, without liver metastasis, without history of previous EGFR target therapy, and former smokers; however, the survival benefit was not statistically significant. Partially, we speculated this was because of the small sample size in our study. Previous studies showed that smoking, wild-type *EGFR*, and absence of liver metastasis predicted longer PFS and increased ORR in NSCLC patients treated with anti-PD-1/PD-L1 antibody (37–39), while acquired resistance to previous EGFR-TKI promoted immune escape (40). Furthermore, in patients who respond to anti-PD-1/PD-L1 antibodies, anti-PD-1/PD-L1 antibodies seem to have durable antitumor efficacy (41). In our study, the failure to demonstrate significant prolongation of PFS and OS was probably due to the small sample size and the short follow-up.

In this study, treatment with anlotinib—either alone or in combination with other therapies—was generally well tolerated. Similar with previous clinical trial and real-world studies, the most common adverse were fatigue, hypertension, hand-foot syndrome and oral mucositis (11–14). In addition, we indicated that hoarseness was also frequently observed in our sample. Fatal bleeding has been reported in other anti-angiogenesis drugs, such as sunitinib, sorafenib and bevacizumab (42). In our study, only 2 patients with mild alimentary tract hemorrhage or hemoptysis were reported and the bleeding stopped after anlotinib was discontinued. No additional adverse event was seen in patients receiving anlotinib plus anit-PD-1/PD-L1 antibodies. Thus, the combination of anlotinib with anit-PD-1/PD-L1 antibodies appears to be safe in NSCLC.

This study has some limitations. First, this was a retrospective analysis of data of a small sample from a single center, and so bias is inevitable and the sample size is not sufficient to obtain statistical difference. Thus, future study with lager sample size is needed. Second, PD-L1 expression is considered as the utmost predictive biomarker for NSCLC patients treated with anti-PD-1/PD-L1 agents (43–45), while the PD-L1 status was not obtained in this study. Third, the follow-up period was short; longer follow-up is necessary to evaluate the effect of anlotinib plus immunotherapy on OS.

In conclusion, anlotinib appears to be well-tolerated and effective in patients with NSCLC. Brain metastasis was found to be an independent predictor of PFS in NSCLC patients receiving anlotinib with or without other therapies. Addition of immunotherapy to anlotinib may prolong PFS in patients with adenocarcinoma, wild-type *EGFR*, stage IV disease, no liver metastasis, exsmoker status, two or more previous treatment lines, and no previous VEGF or EGFR target therapies. Further large prospective studies are needed to confirm our findings. Future studies should also attempt to identify predictive biomarkers that could help identify patients likely to benefit from the combination of anlotinib plus immunotherapy.

## Data Availability Statement

The original contributions presented in the study are included in the article/supplementary material. Further inquiries can be directed to the corresponding authors.

## Ethics Statement

The studies involving human participants were reviewed and approved by Ethics Committee of the General Hospital of Chinese PLA (Medical Ethics Committee of General Hospital of Chinese PLA No. S2018-092-01). The ethics committee waived the requirement of written informed consent for participation.

## Author Contributions

Conception/design: YH, QX; Collection of assembly of data: BQ, BY, QS, YW, SZ; CT evalutation: QX, BQ, YH; Data analysis and interpretation: QX, BQ, LX; Manuscript writing: QX LX; Final approval of manuscript: All authors. All authors contributed to the article and approved the submitted version.

## Conflict of Interest

The authors declare that the research was conducted in the absence of any commercial or financial relationships that could be construed as a potential conflict of interest.

## Publisher’s Note

All claims expressed in this article are solely those of the authors and do not necessarily represent those of their affiliated organizations, or those of the publisher, the editors and the reviewers. Any product that may be evaluated in this article, or claim that may be made by its manufacturer, is not guaranteed or endorsed by the publisher.
